# Rhizosphere melatonin application reprograms nitrogen-cycling related microorganisms to modulate low temperature response in barley

**DOI:** 10.3389/fpls.2022.998861

**Published:** 2022-10-06

**Authors:** Miao Jiang, Fan Ye, Fulai Liu, Marian Brestic, Xiangnan Li

**Affiliations:** ^1^ Key Laboratory of Mollisols Agroecology, Northeast Institute of Geography and Agroecology, Chinese Academy of Science, Changchun, China; ^2^ Key Laboratory of Agricultural Soil and Water Engineering in Arid and Semiarid Areas, Ministry of Education of China, Northwest A & F University, Yangling, China; ^3^ College of Advanced Agricultural Sciences, University of Chinese Academy of Sciences, Beijing, China; ^4^ Faculty of Science, Department of Plant and Environmental Sciences, University of Copenhagen, Tåstrup, Denmark; ^5^ Department of Plant Physiology, Slovak Agricultural University, Nitra, Slovakia; ^6^ Department of Botany and Plant Physiology, Czech University of Life Sciences Prague, Prague, Czechia; ^7^ Chinese Academy of Science (CAS) Engineering Laboratory for Eco-agriculture in Water Source of Liaoheyuan, Chinese Academy of Science, Changchun, China

**Keywords:** melatonin, low temperature, *Hordeum vulgare*, microbial diversity, nitrogen cycling

## Abstract

Rhizospheric melatonin application has a positive effect on the tolerance of plants to low temperature; however, it remains unknown whether the rhizosphere microorganisms are involved in this process. The aim of this study was to investigate the effect of exogenous melatonin on the diversity and functioning of fungi and bacteria in rhizosphere of barley under low temperature. The results showed that rhizospheric melatonin application positively regulated the photosynthetic carbon assimilation and redox homeostasis in barley in response to low temperature. These effects might be associated with an altered diversity of microbial community in rhizosphere, especially the species and relative abundance of nitrogen cycling related microorganisms, as exemplified by the changes in rhizosphere metabolites in the pathways of amino acid synthesis and metabolism. Collectively, it was suggested that the altered rhizospheric microbial status upon melatonin application was associated with the response of barley to low temperature. This suggested that the melatonin induced microbial changes should be considered for its application in the crop cold-resistant cultivation.

## Introduction

Melatonin (*N*-acetyl-5-methoxytryptamine) is an important signal molecule regulating various physiological processes in plants under abiotic stress, including scavenging reactive oxygen species (ROS) as an antioxidant (Arnao and Hernández-Ruiz, 2009), activating antioxidant defense systems (Tan et al., 2012), modulating the expression of stress response genes (Li et al., 2021), and regulating photosynthetic carbon assimilation (Li et al., 2016). Though the original sites of melatonin biosynthesis are mitochondria and chloroplasts in higher plants (Tan and Reiter, 2019), both foliar and rhizospheric melatonin application has been shown to enhance the tolerance of plants to abiotic stress, such as low temperature and drought (Li et al., 2016; Nawaz et al., 2016). For instance, exogenous melatonin increases the tolerance to low temperature of bermudagrass by enhancing endogenous melatonin level and antioxidant enzyme activities (Fan et al., 2015). Spaying melatonin benefited the growth of barley plant under low temperature by promoting photosynthetic carbon assimilation, activating ROS scavenging capacity and optimizing carbohydrate metabolism (Zhao et al., 2015; Li et al., 2016). In addition to a direct effect on root and shoot physiology, rhizospheric melatonin application could also affect some other processes in soil, such as rhizospheric microbial status, which may play key roles in the process of melatonin-induced stress tolerance.

The rhizosphere is the home of an overwhelming number of microorganisms (Philippot et al., 2013), where the soil microorganisms interact most intensely with plant roots, establishing beneficial associations which could mitigate the adverse effects of abiotic stress on plant (Toju et al., 2018). For example, microbial populations that thrive when exposed to abiotic stress, known as “defense biomes”, can benefit plant stress tolerance (Liu et al., 2020). *A. chroococcum* has favorable effects on *Dodonaea viscosa* seedlings, resulting in improved plant growth and seed germination under salinity stress (Yousefi et al., 2017). *Pseudomonas* and *Mesorhizobium* strains assist to improve growth and symbiotic performance of liquorice (*Glycyrrhiza uralensis* Fish.) under abiotic stress (Egamberdieva et al., 2016). Plant-associated microbiomes play a role in determining plant fitness (Goh et al., 2013; Xu et al., 2018) and a number of microbial members have important functions as partners in the environmental adaptation and metabolisms in plants, such as enhanced nutrient uptake, nitrogen fixation and defense responses (Bano et al., 2021). Therefore, the species and abundance of rhizosphere microorganisms are closely related to plant growth and induction of stress tolerance (Mendes et al., 2013; Geng et al., 2018). In addition, as one of the most active components in the soil ecosystem, microorganisms drive the cycles of carbon, nitrogen, phosphorus and sulfur in soil, which also directly modulates plant growth and their responses to abiotic stress (Koide et al., 2011; Eisenhauer et al., 2012; Purahong and Krüeger, 2012).

Soil metabolites, including sugars, amino acids, organic acids, and phenolic compounds, can reflect the changes in important metabolic pathways for soil microbial community (Withers et al., 2020). Metabolic profiles in rhizospheric soils are comprised of a great variety of chemicals, which recruit specific microbial species to form complex relationships with plants (Philippot et al., 2013; Kuzyakov and Razavi, 2019), could be used to characterize soil functional alterations and facilitate the investigation of the link between soil microbial community and soil metabolites (Ding et al., 2021).

Low temperature induces a series of physiological changes in plants, including inactivation of many metabolic enzymes, disturbance of the metabolic regulations, and modifications of the carbohydrate metabolism and photosynthetic properties (Li et al., 2014a; Li et al., 2014b). To explore the relationship between melatonin and microbial diversity and its implications in modulating the response of low temperature stress in barley plant, the diversity of bacterial and fungal microbial, rhizosphere soil metabolome, the enzymatic profiling related to carbohydrate metabolism, redox homeostasis and melatonin metabolism in barley plants were investigated. It was hypothesized that (i) Rhizospherically applied melatonin would significantly alter the diversity of microbial community and re-program the rhizosphere soil metabolites under low temperature; (ii) Melatonin induced the changes of low temperature response in barley would be associated with the nitrogen-cycling related rhizosphere microorganisms.

## Materials and methods

### Plant material and cultivation conditions

The seeds of spring barley cv. Steptoe were sterilized with 80% ethanol and 1% sodium hypochlorite solution and washed with sterile water. Four seeds were sown in each pot (15 cm in diameter and 10 cm in height, with 2 drainage holes) with 1 kg soil. The characteristics of soil used were: pH 7.1, organic carbon 10.98 g kg^−1^, total nitrogen 1.61 g kg^−1^, available nitrogen 141 mg kg^−1^, available phosphorus 62.8 mg kg^−1^, available potassium 147 mg kg^−1^. The plants were grown in the growth chamber at 26°C/20°C (day/night, 22,000 Lux/0 Lux, 12 h/12 h). The relative humidity in the growth chamber was 60% ± 5%. After 21 days of sowing, half of the plants were rhizospherically treated with 1 mmol/L melatonin (MT), while the rest were treated with water as the control (N). The melatonin treatment (100 mL per pot) was applied once every three days for 30 days, and the total melatonin content was 232.28 mg/kg soil. Twelve hours after the last melatonin application, half of the MT plants and the control plants were exposed to a 48-hour low temperature treatment (2 ± 0.5°C). Therefore, four treatments were established: NT_N, normal temperature + water; NT_MT, normal temperature + 1 mmol/L melatonin; LT_N, low temperature + water; LT_MT, low temperature + 1 mmol/L melatonin. Six pots were included in each treatment.

The root samples were gently rinsed several times with tap water, then washed with sterile water, followed by drying on sterilized filter paper. The last fully expanded leaves samples and roots samples were collected immediately after the low temperature treatment and then were snap frozen in liquid nitrogen and stored at -80°C. The roots were carefully taken out of the pots, shaken to remove loosely adhering soil, and then about 1 mm of soil from the roots was retained as rhizosphere soil (Edwards et al., 2015). The rhizosphere soil samples were carefully collected and screened using a 2.0 mm sterile sieve and snap frozen in liquid nitrogen and stored at -80°C.

### Chlorophyll a fluorescence measurement

Three fully expanded leaves from different plants in each treatment were selected for the dark-adapted imaging of maximal photochemical efficiency (F_V_/F_m_) using a FluorCam (FC 800MF, Photon Systems Instruments, Brno, Czech Republic). Just after the end of low temperature treatment, the chlorophyll a fluorescence (OJIP) transient of the same leaves for chlorophyll a fluorescence imaging was measured using a portable fluorometer (Fluorpen FP100, Photon System Instruments, Drasov, Czech Republic).

### Analysis of melatonin metabolism enzyme activity and related metabolites

Concentrations of melatonin and the metabolites related to melatonin synthesis (tryptophan, tryptamine, serotonin, *N*-acetylserotonin) and activities of key enzymes (tryptophan decarboxylase (TDC), tryptamine 5-hydroxylase (T5H), serotonin *N*-acetyltransferase (SNAT), *N*-acetylserotonin *O*-methyltransferase (ASMT), caffeic acid *O*-methyltransferase (COMT)) in roots were detected using enzyme-linked immunosorbent assay (ELISA) kit by an Epoch™ Microplate Spectrophotometer (BioTek Instruments, Inc., Winosky, Vermont, USA). All measurements were determined in triplicate.

### Measurement of antioxidant enzyme activities

According to the protocol of Fimognari et al. (2020), the activities of key antioxidant enzymes in roots, i.e., ascorbate peroxidase (APX), catalase (CAT), cell wall peroxidase (cwPOX), dehydroascorbate reductase (DHAR), glutathione reductase (GR), glutathione S-transferase (GST), monodehydroascorbate reductase (MDHAR), peroxidase (POX), superoxide dismutase (SOD), were measured using an Epoch™ Microplate Spectrophotometer (BioTek Instruments, Inc., Winosky, Vermont, USA) with a semi high-throughput 96-well assay format. The enzyme activities were normalized by fresh weight. The unit of antioxidant enzyme activity was nkat g^-1^ FW. All measurements were determined in triplicate.

### Measurement of carbohydrate metabolism enzyme activities

According to the protocol of Jammer et al. (2015), the activities of 13 key primary carbohydrate metabolism enzymes in leaf and root samples, i.e., ADP-glucose pyrophosphorylase (AGPase), aldolase (Ald), cytoplasmic invertase (cytInv), cell wall invertase (cwInv), fructokinase (FK), glucose-6-phosphate dehydrogenase (G6PDH), hexokinase (HXK), phosphofructokinase (PFK), phosphoglucoisomerase (PGI), phosphoglucomutase (PGM), sucrose synthase (SuSy), UDP-glucose pyrophorylase (UGPase), vacuolar invertase (vacInv), were determined using an Epoch™ Microplate Spectrophotometer (BioTek Instruments, Inc., Winosky, Vermont, USA) with a semi high-throughput 96-well assay format. The enzyme activity was normalized by fresh weight. The unit of carbohydrate metabolism enzyme activity was nkat g^-1^ FW. All measurements were determined in triplicate.

### Concentrations of total soluble sugars, sucrose and reducing sugar

The root samples were harvested and oven-dried to measure the concentrations of total soluble sugar, sucrose and reducing sugar. Concentrations of total soluble sugar and sucrose in dry root samples were measured according to the anthrone reagents method (Fales, 1951). Reducing sugar concentration was measured following our previous methods (Li et al., 2015).

### Determination of urease and nitrate reductase activities and total nitrogen concentrations in soil

Fresh soil samples were oven-dried at 37°C and then sieved by passing through a 50-mesh sieve. The activities of soil urease and nitrate reductase were tested by assay kits (Boxbio Science & Technology Co., Ltd., Beijing, China) using an Epoch™ Microplate Spectrophotometer (BioTek Instruments, Inc., Winosky, Vermont, USA) following the user manual at 630 nm and 520 nm. Soil samples were thoroughly mixed, and the representative sub-samples were extracted immediately using 2 M KCl solution (soil solution ratio: 1:5) and shaken for 1 h on a rotary shaker (180 rev min^-1^), followed by filtration. The concentrations of NH_4_^+^-N, NO_3_^–^N and total nitrogen were measured according to the methods of Shi et al. (2012).

### DNA extraction, sequencing, and microbial community analysis

Soil samples (0.5 g) were used for microbial genomic DNA extraction with the OMEGA Soil DNA Kit (D5625-01) (Omega Bio-Tek, Norcross, GA, USA) following the manufacturer’s instructions for amplification of bacterial 16S V3-V4 and fungal ITS1 regions. The NanoDrop ND-1000 spectrophotometer (Thermo Fisher Scientific, Waltham, MA, USA) and agarose gel electrophoresis were used to measure the quantity and quality of extracted DNAs, respectively. The measurement for each treatment included six biological repeats.

The PCR amplification of the bacterial 16S V3-V4 region was performed using the forward primer 338F (5’- ACTCCTACGGGAGGCAGCA -3’) and the reverse primer 806R (5’- CGGACTACHVGGGTWTCTAAT -3’), the forward primer ITS5F (5’- GGAAGTAAAAGTCGTAACAAGG -3’) and the reverse primer ITS2R (5’- GCTGCGTTCTTCATCGATGC -3’) was for fungal ITS V1 region, simultaneously. Bacteria and fungi microbial genomic DNA had the same PCR components: 5 μL of reaction buffer (5×), 5 μL of GC buffer (5×), 0.25 μL of Fast pfu DNA Polymerase (5U/μL), 2 μL (2.5 mM) of dNTPs, 1 μL (10 uM) of each Forward and Reverse primer, 1 μL of DNA Template, and 9.75 μL of ddH_2_O.

The PCR reactions of bacteria microbial genomic DNA were applied using the following program: 5 min of denaturation at 98°C, 25 cycles of 30 s at 98°C, 30s for annealing at 52°C, and 1 min for elongation at 72°C, and a final extension at 72°C for 5 min. The amplification of fungi microbial genomic DNA were conducted using the following protocol: 5 min of denaturation at 98°C, 28 cycles of 30 s at 98°C, 30s for annealing at 52°C, 1 min for elongation at 72°C, and a final extension at 72°C for 5 min. The resulted PCR products were purified with Vazyme VAHTSTM DNA Clean Beads (Vazyme, Nanjing, China) and quantified using the Quant-iT PicoGreen dsDNA Assay Kit (Invitrogen, Carlsbad, CA, USA). The Illumina NovaSeq 6000 (Hayward CA USA) was used for paired-end sequenced (2 × 250) with the purified amplicons pooled in equimolar (Personal Biotechnology Co., Ltd, Shanghai, China).

Sequences were then quality filtered, denoised, merged and chimera removed using the DADA2 plugin (Callahan et al., 2016). Sequence data analyses were mainly performed with QIIME2 and R packages (v3.2.0). Unique reads with 100% similarity based on the representative 16S or ITS1 sequences were clustered into ASVs (amplicon sequence variants). Taxonomy was assigned to ASVs using the classify-sklearn naïve Bayes taxonomy classifier in feature-classifier plugin (Bokulich et al., 2018) against the SILVA Release132 (http://www.arb-silva.de) for bacteria, while UNITE Release 8.0 (https://unite.ut.ee/) for fungi Database (Kõljalg et al., 2013). The sequences from the host were filtered from the bacterial ASV table. ASV-level alpha diversity indices, such as Chao1 richness estimator, observed species, Shannon diversity index and Simpson index were calculated using the ASV table in QIIME2 by Kruskal-Wallis test and visualized as box plots. LEfSe (Linear discriminant analysis effect size) (http://huttenhower.sph.harvard.edu/galaxy/root?tool_id=lefse_upload) was applied to detect biomarkers at multiple taxonomical levels with an LDA score threshold >3.5 from phylum to genus. Microbial functions were predicted by MetaCyc databases (https://metacyc.org/) at level 1 and 2.

### Soil metabolomic detection and analysis

Soil samples (50 mg) were extracted with 0.5 mL of acetonitrile: isopropanol: water (3:3:2, v/v/v) mixed solution (-20°C) and then vibrated at 30 Hz for 2 min and ultrasonic for 5 min at room temperature. 0.5 mL of acetonitrile, isopropanol and water (3:3:2, v/v/v) mixed solution (-20°C) were added to the extraction, and then it was ultrasonic for 5 min at room temperature, centrifuged at 10 000 g for 2 min. The supernatant was concentrated to dry by vacuum concentrator. Eighty μL of 20 mg/mL MEOX solution was added for redissolution through vortex vibration for 30 s, and then incubated for 60 min (60°C). 100 μL BSTFA reagent was added into the extraction, and then it was reacted at 70°C for 90 min, 90-100 μL of supernatant were added into the detection bottle after centrifuged at 12 000 g for 3 min. To correct the deviation of analysis results of mixed samples and errors caused by the analyzer itself, quality control (QC) was applied. Twenty μL of samples were mixed into the QC samples, the remaining samples were detected by GC-MS. Gas chromatography was performed to separate the derivatives at a constant flow of 1 mL/min helium. Mass spectrometry was determined by the full-scan method with a range from 75 to 650 (m/z). Metabolites with a VIP value > 1.0 and p-value < 0.05 were selected as the ones significantly affected by the treatments. A correlation heatmap was presented to show the correlation between differential metabolites and biomarkers of bacterial and fungal microbial communities.

### Univariate statistical analysis

All data were firstly tested for homogeneity of variance and then subjected to one-way ANOVA to detect significant differences at *P* < 0.05 level. All data were subjected to Duncan-test to determine statistical differences using the SPSS 22.0 (SPSS Inc., Chicago, IL, USA).

## Results

### Chlorophyll a fluorescence and carbohydrate metabolism enzyme activities in leaves

**Figure 1:**
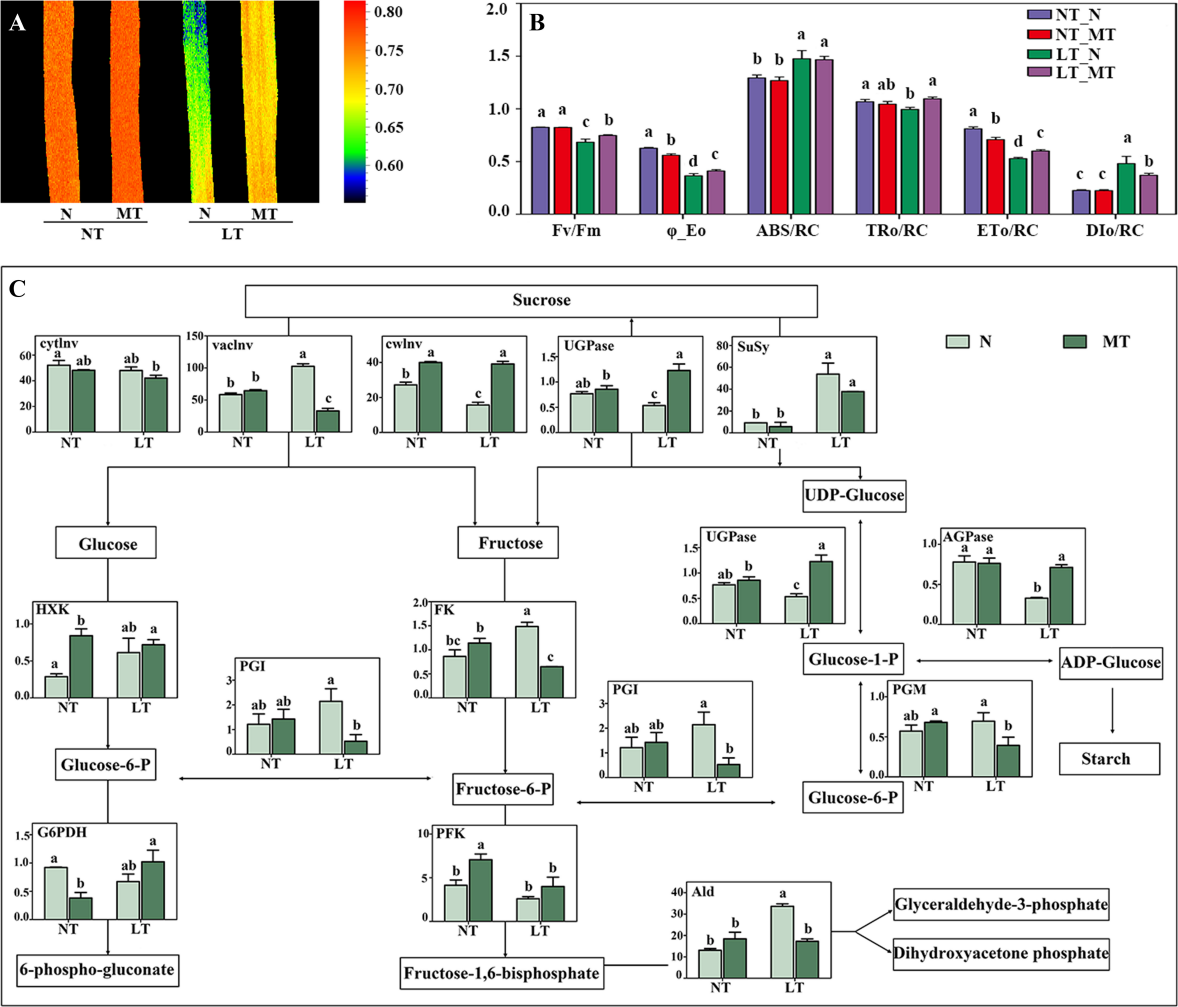
Dark-adapted image of maximum quantum efficiency of photosystem II (Fv/Fm, **A**), chlorophyll a fluorescence parameters **(B)**, and the primary carbohydrate metabolism enzyme activities **(C)** of the last fully expanded leaves as affected by melatonin and low temperature in barley. N, the control; MT, melatonin treatment; NT, normal temperature; LT, low temperature. NT_N, normal temperature control; NT_MT, normal temperature + melatonin treatment; LT_N, low temperature control; LT_MT, low temperature + melatonin treatment. Data are presented as mean ± SE (n = 8 in panel **(B)**; n = 3 in panel **(C)**). Different small letters represent significant differences at *P* < 0.05. The unit of carbohydrate metabolism enzyme activity is nkat g^−1^ FW. φ_E_o_, quantum yield for PSII electron transport; ABS/RC, absorption flux per RC (reaction center); DI_o_/RC, dissipated energy flux per RC (at t=0). AGPase, ADP-glucose pyrophosphorylase; Ald, aldolase; cytInv, cytoplasmic invertase; cwInv, cell wall invertase; ET_o_/RC, electron transport flux per RC (at t=0); FK, fructokinase; Fructose-6-P, fructose-6-phosphate; Glucose-1-P, glucose-1-phosphate; Glucose-6-P, glucose-6-phosphate; G6PDH, glucose-6-phosphate dehydrogenase; HXK, hexokinase; PFK, phosphofructokinase; PGI, phosphoglucoisomerase; PGM, phosphoglucomutase; SuSy, sucrose synthase; TR_o_/RC, trapped energy flux per RC (at t=0); UGPase, UDP-glucose pyrophorylase; vacInv, vacuolar invertase.

The dark-adapted images of Fv/Fm in barley leaves showed that low temperature decreased significantly the Fv/Fm value compared with the normal temperature control (**Figures 1A, B**). However, Fv/Fm of melatonin treated plants was significantly higher than that of the control plants under low temperature. Low temperature significantly reduced the quantum yield for photosystem II (PS II) electron transport (φ_Eo), trapped energy flux per reaction center (TRo/RC) and electron transport flux per RC (ETo/RC), while increased the absorption flux per RC (ABS/RC) and dissipated energy flux per RC (DIo/RC), in relation to the normal temperature control. Under low temperature, melatonin treated plants had significantly higher φ_Eo, TRo/RC and ETo/RC than the control plants, while the DIo/RC was significantly lower in LT_MT plants compared with LT_N plants.

The activities of key enzymes involved in carbohydrate metabolism in leaves were significantly affected by the interaction of melatonin and low temperature (**Figure 1C**). In the sucrolytic pathway, low temperature significantly increased the activity of vacInv but decreased the activity of cwInv compared with the normal temperature control. The MT plants had significantly higher cwInv activity while significantly lower vacInv activity than N plants under low temperature. For the pathway of glycolysis, compared with the normal control, low temperature only significantly increased the activities of FK and Ald, while the activities of PGM, PGI, HXK and PFK were not affected. Under low temperature, MT plants possessed significantly lower activities of FK, Ald, PGM and PGI than N plants; however, no significant difference was found in HXK and PFK activities between MT and N plants. LT_N plants showed significantly lower activity of enzyme related to starch biosynthesis (AGPase) and sucrose biosynthesis (UGPase) compared with NT_N plants, while LT_MT plants had significantly higher activities of these two enzymes compared with LT_N plants. For the pentose phosphate pathway, no significant difference was found in G6PDH activity either between NT_N and LT_N plants or between LT_N and LT_MT plants.

### Melatonin synthesis and metabolism, ROS and carbohydrate metabolism in roots

**Figure 2:**
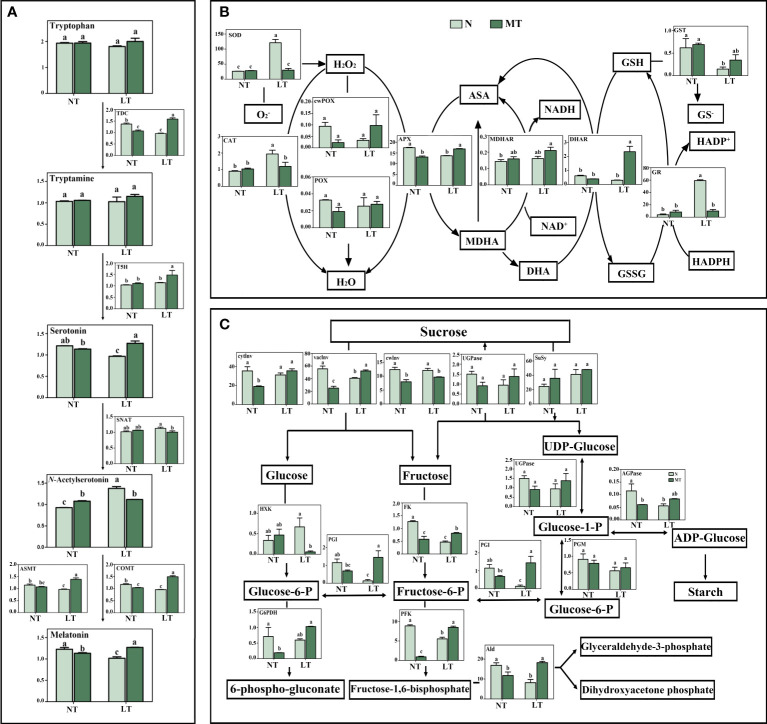
Metabolite concentrations and activities of enzymes involved in melatonin synthesis and metabolism **(A)**, activities of antioxidant enzymes **(B)** and activities of the primary carbohydrate metabolism enzymes **(C)** in roots as affected by melatonin and low temperature in barley. N, the control; MT, melatonin treatment; NT, normal temperature; LT, low temperature. Data are presented as mean ± SE (n = 3). Different small letters represent significant differences at *P* < 0.05. The unit for antioxidant enzyme activity and carbohydrate metabolism enzyme activity is nkat g^-1^ FW. AGPase, ADP-glucose pyrophosphorylase; Ald, aldolase; APX, ascorbate peroxidase; ASA, ascorbic acid; ASMT, *N*-acetylserotonin *O*-methyltransferase, U g^-1^ FW; CAT, catalase; COMT, caffeic acid *O*-methyltransferase, U g^-1^ FW; cwPOX, cell wall peroxidase; cwInv, cell wall invertase; cytInv, cytoplasmic invertase; DHA, dehydroascorbic acid; DHAR, dehydroascorbate reductase; FK, fructokinase; Fructose-6-P, fructose-6-phosphate; G6PDH, glucose-6-phosphate dehydrogenase; Glucose-1-P, glucose-1-phosphate; Glucose-6-P, glucose-6-phosphate; GR, glutathione reductase; GSH, reduced glutathione; GSSG, oxidized glutathione; GST, glutathione S-transferase; HXK, hexokinase; MDHA, monodehydroascorbate; MDHAR, monodehydroascorbate reductase; Melatonin, μg g^-1^ FW; *N*-Acetylserotonin, μg g^-1^ FW; PFK, phosphofructokinase; PGM, phosphoglucomutase; PGI, phosphoglucoisomerase; POX, peroxidase; Serotonin, μg g^-1^ FW; SNAT, Serotonin *N*-acetyltransferase, U g^-1^ FW; SOD, superoxide dismutase; SuSy, sucrose synthase; T5H, Tryptamine 5-hydroxylase, U g^-1^ FW; TDC, Tryptophan decarboxylase, U mg^-1^ FW; Tryptamine, ng g^-1^ FW; Tryptophan, ng g^-1^ FW; UGPase, UDP-glucose pyrophorylase; vacInv, vacuolar invertase.

Low temperature significantly decreased the activities of TDC, ASMT and COMT, while had no significant effect on T5H and SNAT activities in roots, in relation to the normal temperature control (**Figure 2A**). Under low temperature, the activities of most tested enzymes were significantly increased in MT roots, including TDC, T5H, ASMT and COMT, while the SNAT activity was significantly decreased, compared with that under control treatment. The concentrations of serotonin and melatonin were significantly decreased while the *N*-Acetylserotonin concentration was significantly increased in the roots of LT_N plants, in relation to NT_N plants. The concentrations of serotonin and melatonin in MT plants was significantly higher, while the *N*-Acetylserotonin concentration was lower than that of N plants under low temperature. In addition, no significant difference was found in the concentrations of tryptophan and tryptamine among these treatments.

The antioxidant enzyme activity was obviously influenced by the interactive effects of melatonin and low temperature in barley roots (**Figure 2B**). Low temperature significantly increased the activities of SOD, CAT and GR, while reduced significantly the APX and GST activities, compared with the normal temperature control in barley roots. Under low temperature, the activities of APX and DHAR were significantly higher, while activities of SOD, CAT and GR were significantly lower in MT plants compared with N plants.

For the carbohydrate metabolism in roots, low temperature only significantly reduced the activity of vaInv, while had no significant effect on the activities of other enzymes in the sucrolytic pathway (**Figure 2C**). Under low temperature, MT plants had significantly higher vaInv activity while lower cwInv activity than N plants. For the pathway of glycolysis, low temperature significantly reduced the activities of PGI, PFK, Ald and FK, in relation to the normal temperature control. When exposed to low temperature, the MT plants had significantly higher activities of PGI, PFK, Ald and FK, while lower HXK activity than N plants. In addition, the activities of SuSy, PGM and UGPase were not affected by either melatonin or low temperature. Low temperature significantly decreased the concentration of reducing sugar, and slightly reduced the total soluble sugar concentration in roots (**Figure S1**). The concentration of the total soluble sugars was significantly higher in MT roots compared with that in N roots under low temperature.

### Diversity of bacterial and fungal communities

To investigate the influence of low temperature and rhizospheric melatonin application on rhizosphere microbiota compositions, we compared the alpha diversity and the relative abundance of bacterial and fungal communities among treatments. At a threshold of 100% sequence identity and after filtering out the ASVs of chloroplasts and mitochondria from the bacterial ASVs table, 595,3 and 529,42 ASVs were identified in terms of fungi and bacteria, respectively. For fungal communities, 2516, 2215, 2600 and 1832 ASVs had been enriched in NT_N, NT_MT, LT_N and LT_MT, respectively. In addition, in fungal communities, 482 of the common ASVs were enriched among these treatments and 1203, 966, 1257 and 727 of the unique ASVs were enriched in the rhizosphere of NT_N, NT_MT, LT_N and LT_MT, respectively (**Figure S2A**). For bacterial communities, 21874, 19194, 22819 and 15913 ASVs were enriched in NT_N, NT_MT, LT_N and LT_MT. Among them, 377,0 of the common ASVs were enriched in all treatments, while 10212, 8341, 11457 and 7625 of the unique ASVs were enriched in NT_N, NT_MT, LT_N and LT_MT, respectively (**Figure S2B**).

**Figure 3:**
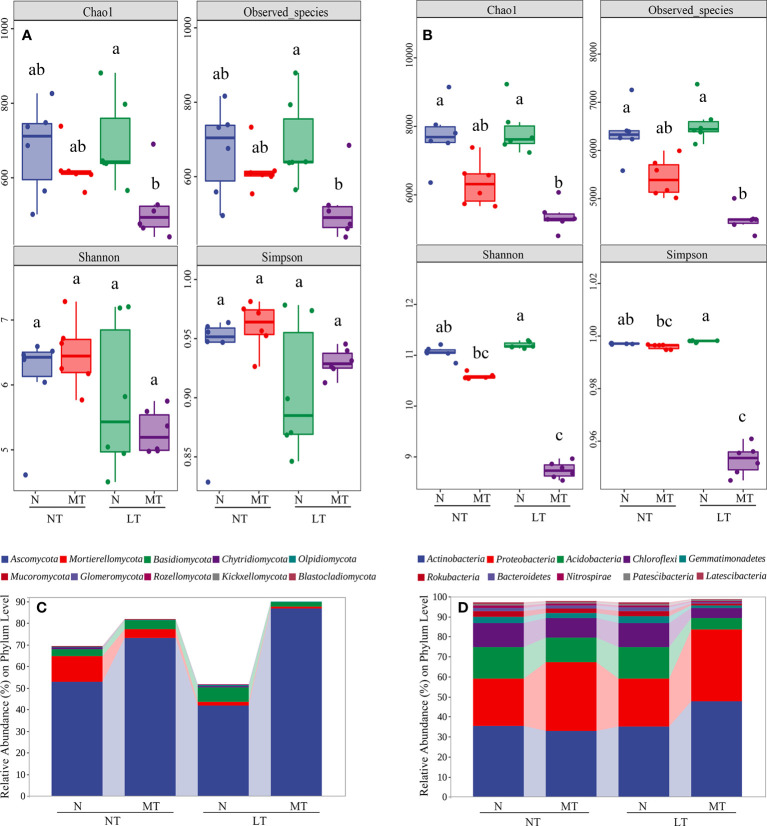
The index of Chao 1, observed species, Shannon and Simpson of fungal **(A)** and bacterial **(B)** microbiota in the rhizosphere at the ASV level and the relative abundance of fungal **(C)** and bacterial **(D)** community at the phylum level as affected by melatonin and low temperature (n = 6). N, the control; MT, melatonin treatment; NT, normal temperature; LT, low temperature.

The alpha diversity of microbial communities as affected by low temperature and rhizospheric melatonin application was further tested. For fungal communities, the Shannon index and Simpson index were similar in the rhizosphere of NT_N, NT_MT, LT_N, and LT_MT at the ASV level (**Figure 3A**). However, significant reductions were found in the alpha diversity index of Chao 1 and observed species in the rhizosphere of LT_MT compared with LT_N. For bacterial communities, no significant difference was found in the alpha diversity between LT_N and NT_N (**Figure 3B**), while rhizospheric melatonin application significantly decreased the alpha diversity indexes (i.e., Chao 1, observed species, Shannon and Simpson) in bacterial community under low temperature.

In this study, the fungal community consisted of 14 phyla, 39 classes, 87 orders, 193 families, 363 genera and 480 species, and the top 10 phyla of fungal community across treatments accounted for 69.51%, 81.85%, 51.60% and 90.03% of the total sequences in NT_N, NT_MT, LT_N and LT_MT, respectively (**Figure 3C**). Ascomycota, Mortierellomycota and Basidiomycota were the dominant fungi with a relative abundance > 1% (at least in one treatment) in fungal community. The proportion of Ascomycota was significantly higher in LT_MT compared with LT_N, and it was the only strain whose abundance increased under low temperature and melatonin among the dominant fungi. In addition, the proportion of Ascomycota was not significantly affected by low temperature. Compared with NT_N, the relative abundance of Mortierellomycota was significantly lower in LT_N. Nonetheless, no significant difference was observed in terms of the proportion of Mortierellomycota in LT_N, in relation to that in LT_MT. For the percentage of Basidiomycota, no significant difference was found between NT_N and LT_N, while that of MT plants was significantly lower than that of the control plants under low temperature.

The community of bacteria consisted of 41 phyla, 112 classes, 262 orders, 420 families, 812 genera and 369 species. The top 10 phyla of bacterial community across treatments accounted for 97.05%, 97.87%, 97.07% and 98.60% of the total sequences in NT_N, NT_MT, LT_N and LT_MT, respectively (**Figure 3D**). Among them, 8 dominant bacteria were found in the top 10 phyla of bacterial community. Actinobacteria and Proteobacteria were the bacteria whose proportion showed an increase trend under low temperature in LT_MT compared with LT_N in dominant bacteria. The other dominant bacteria were Acidobacteria, Chloroflexi, Gemmatimonadetes, Rokubacteria, Bacteroidetes and Nitrospirae, and the percentages of all these six bacteria were significantly lower in LT_MT compared with those in LT_N. The other two bacteria in the top 10 phyla were Patescibacteria and Latescibacteria. All the above bacteria showed no significant difference between NT_N and LT_N, except for Latescibacteria whose percentage was significantly reduced by low temperature.

Under low temperature, *Azotobacter* was not found in the rhizosphere of either NT_N or LT_N (0%), but it was significantly enriched in LT_MT rhizosphere (0.70%) after the melatonin treatment. The percentage of *Azoarcus* was significantly increased in LT_MT rhizosphere (0.51%) compared with that in LT_N (0%). The relative abundance of *Gemmobacter*, *Dechloromonas* and *Ensifer* were significantly increased by melatonin treatment under low temperature. However, all the above five bacteria, which were belong to Proteobacteria, showed no significant difference between NT_N and LT_N. The relative abundances of *Nitrospira* and *Rubrobacter* were significantly lower in LT_MT, compared with LT_N.

### Biomarkers of fungal and bacterial community

**Figure 4:**
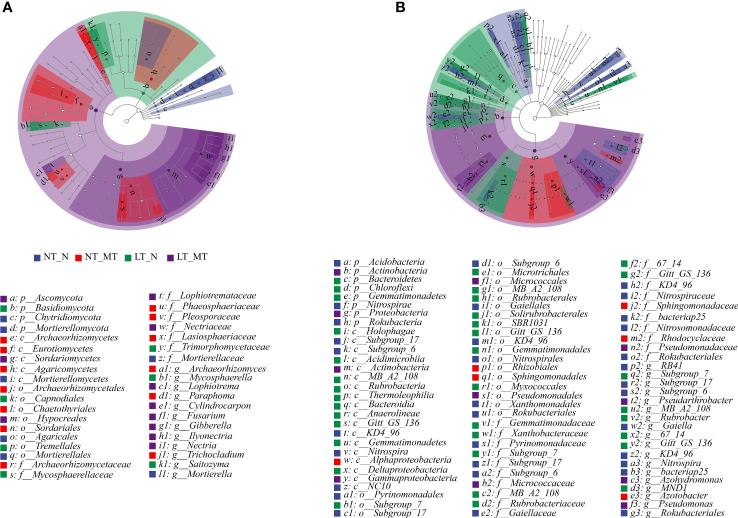
Linear discriminant analysis effect size (LEfSe) of the fungal **(A)** and bacterial **(B)** taxa with a LDA score > 3.5 under low temperature and melatonin treatment. Cladograms indicate the phylogenetic distribution of microbial lineages. Circles represent phylogenetic levels from phylum to genus (n = 6). NT_N, normal temperature control; NT_MT, normal temperature + melatonin treatment; LT_N, low temperature control; LT_MT, low temperature + melatonin treatment.

Linear discriminant analysis effect size (LEfSe) analysis was applied to identify the microbes as biomarkers with LDA scores (> 3.5) from the level of phylum to genus in fungal (**Figure 4A**) and bacterial (**Figure 4B**) communities. These biomarkers showed significant variations in the relative abundances of the core community and were accompanied by considerable changes in response to environmental disturbances. A total of 38 fungi clades exhibited significant variations in NT_N (2 phyla, 1 class, 2 orders, 1 family and 1 genus), NT_MT (3 classes, 3 orders, 4 families and 3 genera), LT_N (1 phylum, 2 orders, 2 families and 2 genera) and LT_MT (1 phylum, 1 class, 1 order, 2 families and 6 genera).

In total, 85 bacteria clades exhibited significant variations in the rhizosphere of NT_N (3 phyla, 5 classes, 8 orders, 9 families and 8 genera), NT_MT (1 class, 2 orders, 2 families and 1 genus), LT_N (3 phyla, 10 classes, 9 orders, 7 families and 6 genera) and LT_MT (2 phyla, 2 classes, 2 orders, 2 families and 3 genera). The bacteria dominant Acidobacteria, Rokubacteria and Nitrospirae at phylum level were significantly abundant in NT_N, and the biomarkers of Nitrospira at class level, Nitrospirales at order level, *Nitrospiraceae* at family level and *Nitrospira* at genus level belonging to Nitrospirae were also observed in NT_N rhizosphere. Alphaproteobacteria at class, Rhizobiales at order, *Rhodocyclaceae* at family and *Azotobacter* at genus were significantly abundant in NT_MT rhizosphere as biomarkers. The proportions of Bacteroidetes, Gemmatimonadetes and Chloroflexi at phylum level, and *Rubrobacter* at genus level were significantly enriched in LT_N. The bacteria dominant Proteobacteria and Actinobacteria at phylum level were significantly abundant in LT_MT rhizosphere. Within Actinobacteria, Gammaproteobacteria at class, Pseudomonadales at order and *Pseudomonadaceae* at family were significantly abundant in the NT_MT rhizosphere.

### Prediction of functional composition in the microbial community

**Figure 5:**
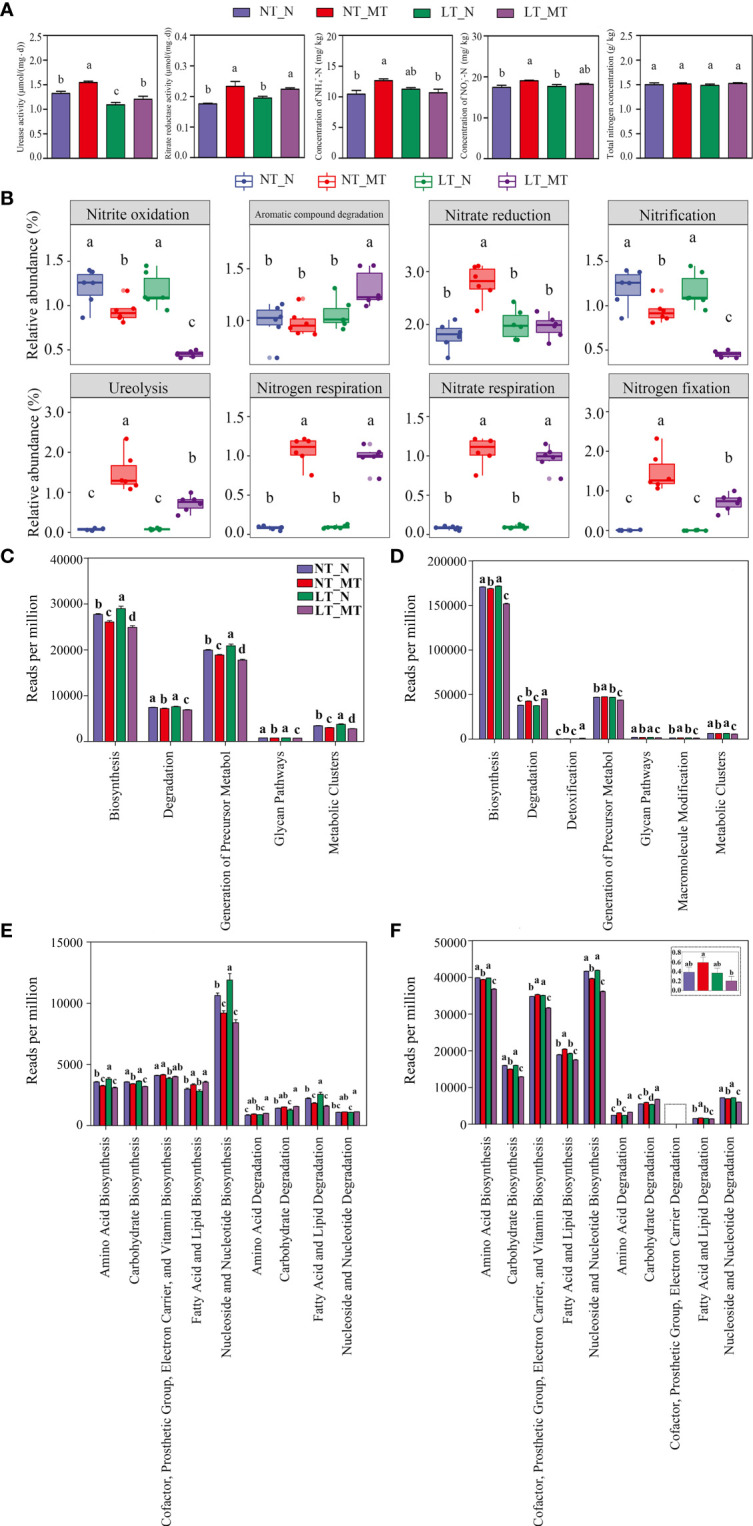
Activities of urease and nitrate reductase and concentrations of NH_4_^+^-N, NO_3_^–^N and total nitrogen in rhizosphere soil and relative abundance of rhizosphere microorganisms with different functions under low temperature and melatonin treatment. Activities of urease and nitrate reductase and concentrations of NH_4_^+^-N, NO_3_^–^N and total nitrogen in rhizosphere soil among different treatments **(A)**. Prediction of the functional composition in the microbial community according to FAPROTAX database **(B)**. Fungal **(C, E)** and bacterial **(D, F)** microbiota at the level 1 and the level 2 according to MetaCyc database, respectively. NT_N, normal temperature control; NT_MT, normal temperature + melatonin treatment; LT_N, low temperature control; LT_MT, low temperature + melatonin treatment. Data are presented as mean ± SE (n = 6). Different small letters represent significant differences at *P* < 0.05.

The activities of urease and nitrate reductase were significantly increased in the rhizosphere soil by rhizospheric melatonin application regardless of temperature treatments (**Figure 5A**). The concentrations of NH_4_^+^-N and NO_3_^–^N were significantly increased by melatonin treatment at normal temperature. In addition, no significant difference was found in the total nitrogen concentration among these treatments.

The prediction of functional composition by FAPROTAX indicated that 7 pathways related to the nitrogen cycle were observed among the 8 most abundant pathways (**Figure 5B**). Low temperature had no effect on the relative abundance of ASVs in any pathway. However, the relative abundance of ASVs related to the nitrogen cycle was significantly increased (i.e., ureolysis, nitrogen respiration, nitrate respiration and nitrogen fixation), while that in the pathways related to nitrite oxidation and nitrification was decreased in LT_MT compared with that in LT_N.

By predicting the functional composition of the microbial community with the sequencing of bacteria and fungi using PICRUSt2, it was found that the major fungal community was enriched in the biosynthesis pathway, degradation processes, generation of precursor metabol, glycan pathways and metabolic clusters (**Figure 5C**); while the bacterial community was enriched in the biosynthesis pathway, degradation processes, detoxification, generation of precursor metabol, glycan pathways, macromolecule modification and metabolic clusters (**Figure 5D**) under these treatments in the level 1 of the MetaCyc database. The biosynthesis pathways were significantly enriched under low temperature in fungal community, while the pathway of degradation was not affected. Nonetheless, for bacterial community, no significant different was observed in LT_N compared with NT_N in the above two pathways. Rhizospheric melatonin application significantly decreased the reads in the fungal community under low temperature in the pathways of biosynthesis and degradation, whereas it was significantly decreased the reads in the pathway of biosynthesis while increased the reads in the pathway of degradation in the bacterial community. In addition, the reads related to the pathways of amino acid biosynthesis was significantly increased in LT_N compared with NT_N in the fungi microbial community. Under low temperature, the reads were significantly enriched in the pathway of amino acid biosynthesis in LT_N, compared with LT_MT, in level 2 in both fungal (**Figure 5E**) and bacterial (**Figure 5F**) communities; however, an opposite trend was found in the pathway of amino acid degradation.

### Correlation between metabolites and biomarkers of microbial communities in rhizosphere soil

**Figure 6:**
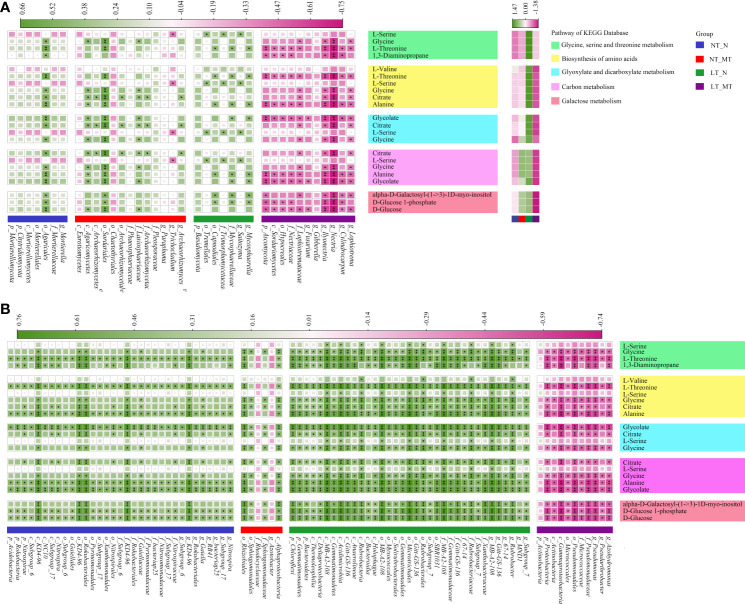
Correlations between biomarker taxon and differential metabolites in rhizosphere for fungal **(A)** and bacterial **(B)** under low temperature and melatonin treatment. The spearman rank correlation coefficients and the corresponding *P*-values were calculated based on the analyses of the microbiota whose LDA score > 3.5 with phylogenetic levels from phylum to genus and the metabolites enriched in the top 5 functions of KEGG pathway in rhizosphere. The proportion of the square and the darkness of the color in each box corresponds to the r^2^ value according to the legend. NT_N, normal temperature control; NT_MT, normal temperature + melatonin treatment; LT_N, low temperature control; LT_MT, low temperature + melatonin treatment. * and ** indicate significance at *P* < 0.05 and *P* < 0.01. Heatmap of metabolite concentration under low temperature and melatonin treatment is shown in the right part of panel **(A)**. The difference of abundance for metabolites among these treatments is deviation standardization with Z-score and converted to a color scale. Increase and decrease in abundance is indicated in the colored bar with pink and green.

The soil metabolites pool, which composed of both plant-secreted metabolites and exogenous metabolites from the microbial community, was significantly changed by low temperature and melatonin (**Figure 6**). Using GC-MS based non-target metabolomics, a total of 104 metabolites were identified and semi-quantified in soil samples. Furthermore, using a statistical threshold of *P* < 0.05 and VIP (variable importance in the projection) > 1, 63 differential metabolites were found in the group of LT_N vs LT_MT. For pathway analysis of the identified differential metabolites using the KEGG database, the *P* < 0.05 and IF (impact factor) > 0.04 were set as threshold values. The TOP 5 pathways, including 11 specific metabolites were divided into two categories: 2 pathways related to amino acid synthesis and metabolism (glycine, serine and threonine metabolism, biosynthesis of amino acids) and 3 carbon metabolism related pathways (glyoxylate and dicarboxylate metabolism, carbon metabolism and galactose metabolism). The metabolites could be annotated into different pathways: L-serine, glycine, L-threonine and 1,3-diaminopropane were annotated into the pathway of glycine, serine and threonine metabolism (*P* = 0.01, IF = 0.43). The amino acids biosynthesis pathway (*P* = 0.01, IF = 0.08) including L-valine, L-threonine, L-serine, glycine, citrate and alanine. Glycolate, citrate, L-serine and glycine belonged to the glyoxylate and dicarboxylate metabolism pathway (*P* = 0.01, IF = 0.07). Citrate, L-serine, glycine, alanine and glycolate were annotated into the pathway of carbon metabolism (*P* = 0.03, IF = 0.05). The alpha-D-galactosyl-(1->3)-1D-myo-inositol, D-glucose 1-phosphate and D-Glucose belonged to the galactose metabolism pathway (*P* = 0.03, IF = 0.05).

The heatmap presented the relationship between biomarkers of fungal community and significantly varied metabolites (**Figure 6A**). The dominant phylum Ascomycota showed significantly negative correlations with L-threonine, 1,3-diaminopropane, alanine, glycolate, alpha-D-galactosyl-(1->3)-1D-myo-inositol, D-glucose 1-phosphate and D-glucose. The biomarkers of Nitrospira, Nitrospirales, *Nitrospiraceae*, *Nitrospira* and Nitrospirae all showed significantly positive correlations with L-threonine, 1,3-diaminopropane, citrate, alanine, glycolate, D-glucose 1-phosphate and D-glucose (**Figure 6B**). The Chloroflexi, Gemmatimonadetes and Bacteroidetes, Alphaproteobacteria, Rhizobiales showed significantly positive correlation with glycine, L-threonine, 1,3-diaminopropane, citrate, alanine, glycolate, alpha-D-galactosyl-(1->3)-1D-myo-inositol, D-glucose 1-phosphate and D-glucose. In addition, Proteobacteria, Gammaproteobacteria, Pseudomonadales and *Pseudomonadaceae* showed significantly negative correlations with those metabolites. *Rubrobacter* significantly positive correlations with all metabolites except for 1,3-diaminopropane.

## Discussion

### Rhizospheric melatonin application enhanced the low temperature tolerance by maintaining a better photosynthetic carbon assimilation

Photosynthesis is a highly sensitive process to low temperatures (Tan et al., 2008). In the present study, low temperature reduced the quantum yield of PS II, while rhizospheric melatonin application alleviated this damage induced by low temperature. φ_Eo reflects the highest quantum yield for PS II electron transport, while TRo/RC and ETo/RC reflect the trapped energy flux per reaction center and the electron transport flux per RC, respectively (Chen et al., 2014). Here, these parameters were all significantly enhanced by rhizospheric melatonin application, indicating that melatonin helped the plant to maintain a higher photosynthetic efficiency by alleviating the damage to the photosynthetic apparatus under low temperature.

Carbohydrate synthesis and catabolism are closely related to abiotic stress tolerance in plants (Jammer et al., 2015). Here, the carbohydrate metabolism in both leaves and roots was affected by the rhizospheric melatonin application. For leaves, in the sucrolytic pathway, the melatonin treated plants had a lower vacInv activity while a higher cwInv activity than the control plants under low temperature, which was contrary to the trends in roots. As a soluble acidic invertase, vacInv determines the sucrose amount stored in vacuole and its remobilization for metabolism (Roitsch and González, 2004); whereas cwInv maintains the extracellular sucrose gradient by breaking down sucrose (Jammer et al., 2015). Thus, the changes of these two invertases may result in the fluctuation of sucrose concentration. For the pathway of glycolysis, lower activities of FK, PGI and Ald in MT leaves in relation to the control plants under low temperature indicated that the conversions of fructose and glucose-6-phosphate to fructose-6-phosphate and fructose-1,6-bisphosphate to glyceraldehyde-3-phosphate and dihydroxyacetone phosphate were both depressed. However, in relation to those in control, the significantly enhanced activities of PGI, PFK, Ald and FK in MT roots suggested that fructose metabolism and glycolysis flux were regulated by melatonin (Dhatt et al., 2019), which might contribute to the enhanced low temperature tolerance in barley. This was also proved by the higher levels of total soluble sugars in roots of melatonin treated barley.

In plants, melatonin has a common biosynthetic pathway from tryptophan through sequential enzymatic steps, including TDC, T5H, SNAT and COMT/ASMT (Nawaz et al., 2016). Rhizospheric melatonin application significantly increased the activities of all these five enzymes except for SNAT under low temperature, indicating that rhizospheric melatonin application enhanced the endogenous melatonin synthesis, which was consistent with results of Sun et al. (2020). In agreement with the enzyme activities, the serotonin and endogenous melatonin levels could be related to the melatonin induced low temperature tolerance, since serotonin and melatonin are of importance for stress defense and plant growth (Li et al., 2016; Wan et al., 2018). Besides the direct effects of rhizospheric melatonin application, it is hard to rule out the possibility of rhizosphere microorganisms for enhancing the stress tolerance in plants (Pieterse et al., 2014).

### Rhizospherically applied melatonin altered the diversity of microbial community related to nitrogen cycling

The alpha diversity refers to the indicators for richness and diversity of fungal and bacterial species, including Chao 1, observed species, Shannon and Simpson. The indexes of Chao 1 and observed species both represent richness (Chao, 1984), while Shannon and Simpson represent the diversity of microorganisms (Shannon, 1948; Simpson, 1997). Here, two indexes (Chao 1 and observed species) for fungal communities and four indexes (Chao 1, observed species, Shannon and Simpson) for bacterial communities all showed significant differences among treatments, which implied that rhizospherically applied melatonin changed the richness of fungal communities and altered both richness and diversity of bacterial communities under low temperature. Similarly, exogenous melatonin alters the structure of soil bacteria and has a significant effect on bacterial alpha diversity under abiotic stress conditions (Madigan et al., 2019). In addition, exogenous melatonin reduces the Shannon diversity of bacterial community and reprograms the rhizosphere microbial community to modulate the responses of barley to abiotic stress (Ye et al., 2022), which is consistent with our findings. However, the release of secondary metabolism compounds from plants into the soil as root exudates may also have an indirect effect on rhizosphere microbial diversity (Wolfe and Klironomos, 2005; Trivedi et al., 2021).

Also, the compositions of fungal and bacterial communities were modulated by rhizospherically applied melatonin regardless of temperature regimes, where Ascomycota, Mortierellomycota and Basidiomycota were the most dominant phyla in the rhizosphere microorganisms. As the most common and diverse community of eukaryotes, Ascomycota is involved in the decomposition of organic substrates (e.g., dead leaves, wood chips and faeces), and it is the dominant fungal community in organically improved soils (Guo et al., 2018). The significantly higher relative abundance of Ascomycota in LT_MT compared with LT_N indicated that melatonin could help to decompose organic substrates. Meanwhile, Basidiomycota has also been proved to be the main decomposer that plays an important role in transformation of soil nutrients and degrade lignocellulose and organic matter (Yelle et al., 2008). The significant difference in the relative abundance of Basidiomycota between LT_MT and LT_N suggested that melatonin had an important effect on nutrient metabolism in the rhizosphere of barley plants.

Besides fungal communities, some significantly altered bacterial communities with functioning in nitrogen fixation and nutrient cycling were also observed in this study. Under low temperature, MT treatment showed a significantly higher relative abundance of Actinobacteria and Proteobacteria compared with the control. As the dominant bacterium with the highest relative abundance, Actinobacteria is the soil-dwelling organisms with an important role in the turnover of organic matter, nutrient recycling and plant growth (Trujillo et al., 2015). Meanwhile, Actinobacteria is a nitrogen-fixing microorganism that has received wide attentions (Verma et al., 2009). In addition to being involved in nitrogen cycling processes, Actinobacteria from special and extreme habitats probably contain novel taxa and compounds for enhancing their host tolerance to environmental stress (Mesa et al., 2017; Qin et al., 2018). For instance, Actinobacteria act as important players in inhibiting the root growth under infection of pathogens (Bhatti et al., 2017) and is associated with regulating plant abiotic stress response (Xu et al., 2021). For another dominant bacterium, Proteobacteria plays a vital role in nitrogen fixation in the biosphere as well as in the carbon, sulfur and nitrogen cycles (Kersters et al., 2006). It was also reported that Proteobacteria has various functions, including protein and amino acid metabolism, carbohydrate metabolism and energy metabolism (Jiang et al., 2021). The significantly increased relative abundances of Actinobacteria and Proteobacteria indicated that rhizospherically applied melatonin regulated the nutrient recycling, nitrogen-fixing and amino acid metabolism, which might enhance the plant performance under low temperature. Acidobacteria participates in nitrogen metabolism and exopolysaccharide production (Kuramae and de Assis Costa, 2019). The significant changes in the relative abundance of Acidobacteria also proved that melatonin induced changes in the fungal and bacterial communities were closely related to the nitrogen metabolism.

The relative abundances of some other microorganisms associated with the nitrogen-cycling were also significantly changed by melatonin under low temperature. Nitrite oxidation is the main biochemical pathway that produces nitrate, and this process is catalyzed by nitrite oxidoreductase, which is encoded by aerobic nitrite-oxidizing bacteria (Daims et al., 2016), including the dominant phyla Chloroflexi and Nitrospirae in this study. The favorable effects of *Azotobacter* have been reported to provide nutrients to cereals and enhance plant growth (Sohal et al., 1998; Baghaie and Aghili, 2021). The genus *Azoarcus* can secrete auxin (Rasul et al., 1998) and promote plant growth (Mehnaz et al., 1998; Raittz et al., 2021). *Dechloromonas* includes genes for dozens of metabolisms, such as nitrogen fixation protein, nitrogen regulatory protein, has the ability to metabolize nitrogen, including nitrogen fixation, denitrification and dissimilatory nitrate reduction (Zhang et al., 2021). The significantly increased in the relative abundance of *Azotobacter*, *Azoarcus* and *Dechloromonas* in MT treatment further proved that melatonin induced changes in microorganism under low temperature were closely related to the nitrogen metabolism, which was beneficial to the plant growth. The common bacterium genus *Nitrospira* was restricted to oxidize nitrite to nitrate in the pathway of nitrification (Stein and Klotz, 2017). The altered compositions in the nitrogen-cycling related bacterial and fungal communities suggested that rhizospherically applied melatonin might actively coordinate the microbial community to modulate soil nutrients for optimal plant growth under low temperature.

Nitrogen-transforming microorganisms are generally classified according to one of the processes, such as nitrification, denitrification, nitrogen fixation, et al. (Kuypers et al., 2018). Notably, it has been observed that some pathways were related to the nitrogen cycle according to the FAPROTAX database in bacterial communities. The significant decrease in the relative abundance of nitrite oxidation and nitrification while the significant increase in that of ureolysis, nitrogen respiration, nitrate respiration and nitrogen fixation were observed regardless of temperature regimes, which could be ascribed to the melatonin application. The activity of soil urease and nitrate reductase were significantly enhanced by melatonin, indicating that melatonin promoted the nitrogen cycling in soil. The concentrations of NH_4_^+^-N and NO_3_^–^N were not affected by melatonin under low temperature; that might be due to the complexity of nitrogen metabolism changes induced by melatonin in the barley rhizosphere. It has been well known that diverse microorganisms can fix dinitrogen gas and denitrify simultaneously (Stein and Klotz, 2017). Furthermore, by predicting the functional composition with the reads of bacterial and fungal communities using the MetaCyc database in level 2, it was found that the reads related to amino acid biosynthesis was significantly decreased, while that related to amino acid degradation was significantly increased by rhizospherically applied melatonin, which also proved this result.

### Melatonin induced changes of metabolites in synthesis and metabolism of amino acids pathway in response to the low temperature tolerance

To verify whether the changes of microbial diversity affect the rhizosphere micro-environment of barley plants, metabolites in rhizosphere soil were detected in this study. The pathway enrichment analysis with the KEGG database was conducted to elucidate the specific changes in rhizosphere metabolic processes. The metabolism of glycine, serine and threonine and biosynthesis of amino acids were the top two altered pathways in the rhizosphere soil, which were both amino acid synthesis and metabolic related pathways. Most of the biomarkers of bacteria and fungi showed significantly positive correlations with the altered metabolites included in these two pathways. However, all the biomarkers showed significantly negative correlations with the metabolites related to amino acid synthesis and metabolism in response to low temperature and melatonin treatments. Soil amino acids can be rapidly decomposed by the microorganisms within a few hours and are an important part of soil nitrogen cycling (Ma et al., 2021). This suggested that rhizosphere microorganisms might accelerate the decomposition of amino acids in the soil to regulate the soil nitrogen cycle in this case. Modulations in certain metabolic pathways might be a key strategy for nitrogen-cycling related microbial communities to help plants adapt to environmental stress.

## Conclusion

Rhizospheric melatonin application promoted the low temperature tolerance in barley, as exemplified by higher photosynthetic carbon assimilation and better redox homeostasis. The melatonin treatment obviously changed in the diversity of microbial community in rhizosphere of barley, especially the species and relative abundance of nitrogen cycling-related microorganisms, which was also related to the changes in rhizosphere soil metabolites in the pathways of amino acid synthesis and metabolism. The altered rhizospheric microbial status were associated with the promotion of the performance of both roots and shoots in barley exposed to low temperature, which might be one of the main reasons for modulate the response of barley to low temperature. Thus, the rhizospheric melatonin application induced low temperature tolerance in barley may be associated with the nitrogen-cycling related rhizosphere microorganisms. These results have important implications for understanding the role of rhizospheric melatonin in regulation of plants to improve low temperature resistance.

## Data availability statement

The original contributions presented in the study are publicly available. This data can be found here: NCBI, PRJNA861116.

## Author contributions

MJ: Conceptualization, Methodology, Writing - original draft. FY: Visualization, Software. FL: Conceptualization, Methodology. MB: Software, Validation. XL: Conceptualization, Methodology, Writing - review & editing. All authors contributed to the article and approved the submitted version.

## Funding

This research was funded by the Strategic Priority Research Program of the Chinese Academy of Sciences (XDA28020400), National Natural Science Fund for Excellent Young Scholars (31922064), CAS Pioneer Hundred Talents Program (C08Y194), the Science & Technology Development Program of Jilin Province (20190201118JC; 20210402036GH) and Danmarks Frie Forskningsfond (0217-00084B).

## Conflict of interest

The authors declare that the research was conducted in the absence of any commercial or financial relationships that could be construed as a potential conflict of interest.

## Publisher’s note

All claims expressed in this article are solely those of the authors and do not necessarily represent those of their affiliated organizations, or those of the publisher, the editors and the reviewers. Any product that may be evaluated in this article, or claim that may be made by its manufacturer, is not guaranteed or endorsed by the publisher.
