# Supplementary material for: Rhizosphere melatonin application reprograms nitrogen-cycling related microorganisms to modulate low temperature response in barley
Source: Front Plant Sci. 2022 Oct 6;13:998861. doi: 10.3389/fpls.2022.998861 (PMC9583915; doi:10.3389/fpls.2022.998861)

Fig. S1 Concentrations of total soluble sugars, sucrose and reducing sugar in barley roots as affected by melatonin and low temperature. The unit is mg/g. Different small letters represent significant differences at *P* < 0.05. NT_N, normal temperature control; NT_MT, normal temperature + melatonin treatment; LT_N, low temperature control; LT_MT, low temperature + melatonin treatment.

Fig. S2 Venn diagram demonstrating shared and unique fungal (A) and bacterial (B) taxa enriched in the rhizosphere under different treatments at the ASV level. NT_N, normal temperature control; NT_MT, normal temperature + melatonin treatment; LT_N, low temperature control; LT_MT, low temperature + melatonin treatment.

Fig. S1


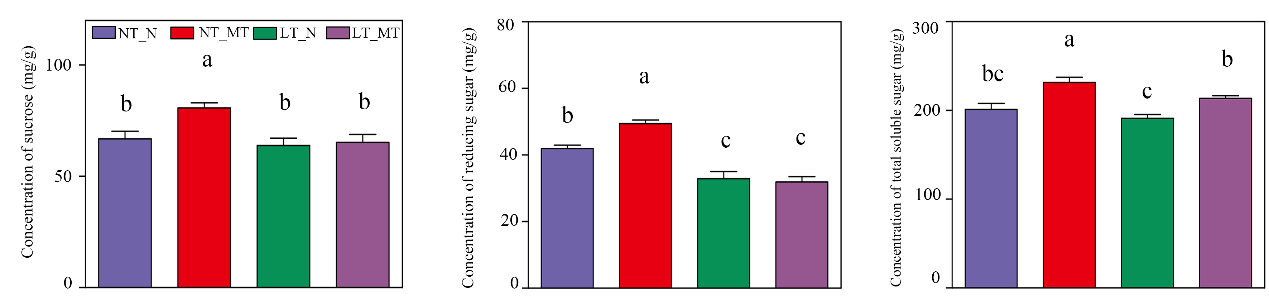


Fig. S2


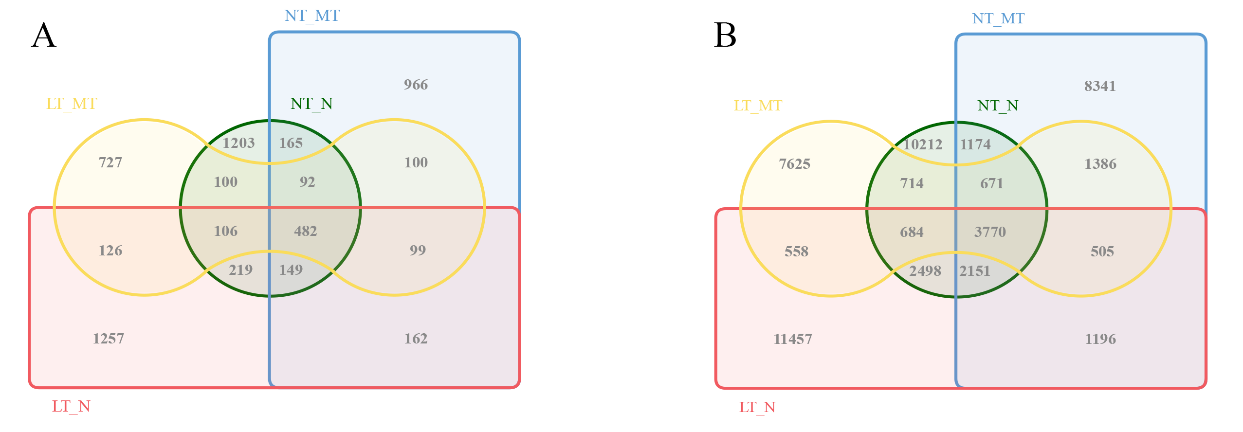

Supplement: Supplementary file 1 [file DataSheet_1.docx]
